# Combustion of raw Camelina sativa oil in CI engine equipped with common rail system

**DOI:** 10.1038/s41598-023-46613-y

**Published:** 2023-11-13

**Authors:** Grzegorz Pawlak, Tomasz Skrzek

**Affiliations:** 1 Military Institute of Armoured and Automotive Technology, Okuniewska 1 Street, 05-070 Sulejówek, Poland; 2 Faculty of Mechanical Engineering, Casimir Pulaski Radom University, Malczewskiego 29 Street, 26-600 Radom, Poland

**Keywords:** Ecology, Environmental sciences, Energy science and technology, Engineering

## Abstract

During the world energy transformation, using some vegetable oils as fuel enables the production of clean and inexpensive energy with the application of the well-known technology of the CI engine. The common rail (CR) fuel system gives many opportunities related to creating an air–fuel mixture and its efficient combustion. Can the system dedicated to diesel oil be used to inject vegetable oil and control its combustion effectively? This paper presents some results of the application of the injection strategy for raw Camelina sativa (CS) oil fueling. The test was conducted on the AVL single-cylinder CI engine equipped with a CR system for engine speed n = 1500 rpm and different excess air coefficients (1.1 < λ < 2.1). The engine parameters and performance obtained for raw CS oil were compared with the results obtained for diesel oil fueling for the same and slightly modified injection strategy. The experiment demonstrated how much the combustion process and its effects differ for these two fuels. In addition, several aspects related to the cultivation and use of Camelina sativa oil as a renewable energy source are presented.

## Introduction

Using relatively cheap vegetable oil from local sources can increase green energy production and reduce long-distance fuel transportation^1^. In this case, a power generator could be a good solution if the engine performance and reliability are high. However, the efficient use of straight vegetable oils as fuel for CI engines remains a challenge. The specific properties of such fuels make creating the air–fuel mixture, including the injection process, which is crucial for the combustion process, significantly different from diesel oil fueling.

Because in most cases where fuel other than diesel oil is used, the injection nozzles remain unchanged, the results of engine fueling with alternative fuel are strongly influenced by modification of the injection process resulting from the properties of this fuel. The comparison of the results to diesel oil fueling gives information which can be used to modify injection parameters or even modify engine construction.

The core differences in the combustion process of different fuels are determined by their ignition delay, which accounts for the physical and chemical properties contributions separately (both usually overlap in time). Lower cetane number and higher flash point, inversely related to fuel volatility, delay the ignition of air/vegetable oil mixture^2,3^. Also, density and even more viscosity profoundly impact the combustion efficiency of CI engines, thus having a notable influence on their expected life^4^. Higher vegetable oil density and viscosity lead to poor atomisation, influencing spray patterns (cone and penetration) and droplet size distribution^5^.

The comparison of conventional fuels and biofuels spray was described in Ref.^6^. The Authors of the paper used Mie scattering and Schlieren imaging techniques to find that the boiling point and density of the fuel significantly impacted the liquid penetration. The experimental results showed that the vapour diffusion and vapour penetration were independent of these parameters^7^. The results presented in Ref.^8^ also confirm that the vapour length is strongly determined by injection pressure. Authors of the paper confirmed that the ambient temperature subtly affects vapour penetration but significantly affects liquid penetration, which is especially important for low-volatility fuels like vegetable oils. The higher boiling temperature delays the evaporation and formation of a combustible mixture. The results presented in Ref.^9^ show that the evaporation of injected biodiesel was suppressed by lower fuel temperature. Its drop from 360 to 300 K caused liquid fuel penetration to lengthen and vapour fuel mass to decrease. The Authors of Ref.^10^ calculated that at high fuel injection pressures and ambient temperature, the evolution of microalgae oil spray was utterly different from diesel oil. For this fuel, the atomisation was worse (the share of larger droplet size in the spray raised by 41–49%), the spray cone angle narrowed by 40–58%, and the distance from the exit of the nozzle to the break-up point was longer by 307–419%.

Recent works like Refs.^11,12^ on flash boiling spray generation by doping high-volatile fuels with typical gasoline or diesel fuels show some benefits to the combustion of premixed combustion engines such as HCCI or RCCI engines. However, the high ambient pressure suppresses the flash boiling vapourisation, so its application or similar phase-changing sprays for non-premixed CI engines is not recommended^7^. Especially when fuels such as raw vegetable oil are used, preheating could physically control ignition and combustion processes in such engines^13^. Vegetable oils are much more reactive to oxygen, so preheating accelerates the oxidation reaction, partially diminishing the role of phenomena that delay the process of creating a flammable mixture and thus increase the self-ignition delay.

The poorly atomised fuel spray, especially for low-pressure fuel systems, could result in incomplete combustion and increased wetting of the engine's internal surfaces, thereby diluting the engine lubricant. The higher the viscosity, the greater the tendency to form deposits on engine elements. It is crucial for a low-temperature engine run. The low-temperature behaviour of vegetable oil could be improved by using suitable pour-point depressants or by blending with diesel oil or kerosene-type fuel (e.g. JP-8)^14^.

Common rail system designed and applied for diesel oil gives many opportunities to control combustion and its effects^15,16^. The high-pressure circuit in the system raises the fuel’s temperature, contributing to the modification of fuel properties, including kinematic viscosity reduction^17,18^. The drop of oil viscosity increases the possibility of obtaining a fuel droplet diameter spectrum in the engine cylinder, allowing for their rapid evaporation and the formation of a combustible mixture.

From the point of view of potential benefits connected with raw oil application for bio-energy production, it is interesting to know whether the properties of Camelina sativa, especially kinematic viscosity and their modification in higher temperatures, enable its practical application for CI engines with CR systems. In this context, it is essential to ensure control of the combustion process of the air/fuel mixture.

Since the combustion process of raw Camelina oil in a CI engine equipped with a CR fuel system is not widely analysed in the literature, the work is devoted to controlling combustion by adjusting the injection strategy, which takes into account fuel properties. The important part of the experiment was to show the difference in the combustion process between Camelina sativa and diesel oil. The experiment was to check whether it is possible to implement an efficient injection strategy of Camelina sativa oil, enabling the CI engine to achieve high engine performance and high thermal efficiency.

## Camelina sativa as an energetic plant

The share of electricity produced from biomass in European Union countries is only a few per cent. In most European countries, this share in the current energy production process is 1–2%. The exception is Germany, which reaches 8–10%^19^. In all these countries, the potential of biomass production and existing power generation installation is much higher, and it is possible to increase it by utilisation of new renewable fuels in the local facilities. Using biomass for energy production can reduce CO_2_ emissions in the long run by creating a closed loop of circulation^20^. During the transition to the global use of hydrogen and utilisation of solar and wind energy on a massive scale, vegetable fuels enable clean and cheap energy generation using well-known power generation technology based on IC engines. Such an approach could contribute to realising the European strategy called “Fit for 55”, assuming a reduction of greenhouse gas emissions in 2030 by 55% compared to 1990^21^.

Camelina sativa as a potential energetic plant is widely described in the literature, but its practical usage in this field is still untapped^22,23^. It is one of the eleven other plants (such as a highland twig, canary reed, and willow) that can be cultivated on marginal agricultural land in Europe, North America and Asia. Such land, affected by low temperatures, allows for industrial crop cultivation without competing directly with food security or biodiversity conservation. It is about 8.37 Mha of European agricultural area^24^. The authors of Ref.^25^ analyse the economic aspect of growing Camelina sativa in the western United States, concluding that it can offset on-farm diesel oil use. As a result, it may increase farm income, diversify rural economic development, and contribute to achieving energy policy goals. Camelina sativa can also contribute to developing the Indian biofuel sector^26^. Three years of adaptability experiments in the warm and dried region of Iran showed tolerance of this crop to abiotic stress such as low temperature, drought and salinity and proved that this crop is suitable for poor soils in this country^27^. CS is a short-season crop. Its growth takes 85 to 100 days^28,29^. It can be harvested with high seed yield and crushed for oil (30–43%)^30,31^ with a lower heating value of about 38 MJ/kg^32^. It is possible to get 1500–3000 kg/ha per year (wet weight)^33^, but genetic modification could increase the amount of oil obtained from seeds^34^. The plant is resistant to common pests and pathogens affecting similar crops. The remaining parts left over from the oil extraction process can be used to produce omega-3-rich animal feed. The heating value of Camelina sativa grey strove is about 15 MJ/kg, and the pomace heating value is 22 MJ/kg^35^, so instead of animal feed or fiberboard, it could be utilised as a source of heat. A high flash point (> 220 °C) allows raw oil to be stored at high temperatures without any fire hazard^36^. Several publications are devoted to biodiesel from Camelina sativa^37–39^ or engine performance fueled with its blends with diesel oil^40–43^. Camelina sativa oil can be converted into biodiesel, and many properties of it meet quality standards like ASTM D6751 or EN 14214. The exception is the iodine number, which is a measure of the unsaturation degree of biodiesel and influences its stability during storage (about 160 (gI/100 g))^44^. Also, parameters like linolenic acid and polyunsaturated methyl ester, viscosity, cetane number, and cold filter plugging point (CFPP) could exceed the allowable ranges^33^. Antioxidants can enhance the oxidation stability of biodiesel, but their presence in biodiesel might change fuel behaviour in the engine^45,46^.

The transesterification of vegetable oils and their additional treatment give high-quality fuel but require consuming significant energy, alcohol and chemicals. It also requires skilled labour and particular equipment investment. It is a time-consuming and expensive process^47^.

Some studies on the direct use of raw Camelina sativa oil as fuel for CI engines were described^32,48,49^. Such an application can be beneficial from the point of view of environmental impact and simplicity in use^50^.

## The experiment

**Figure 1:**
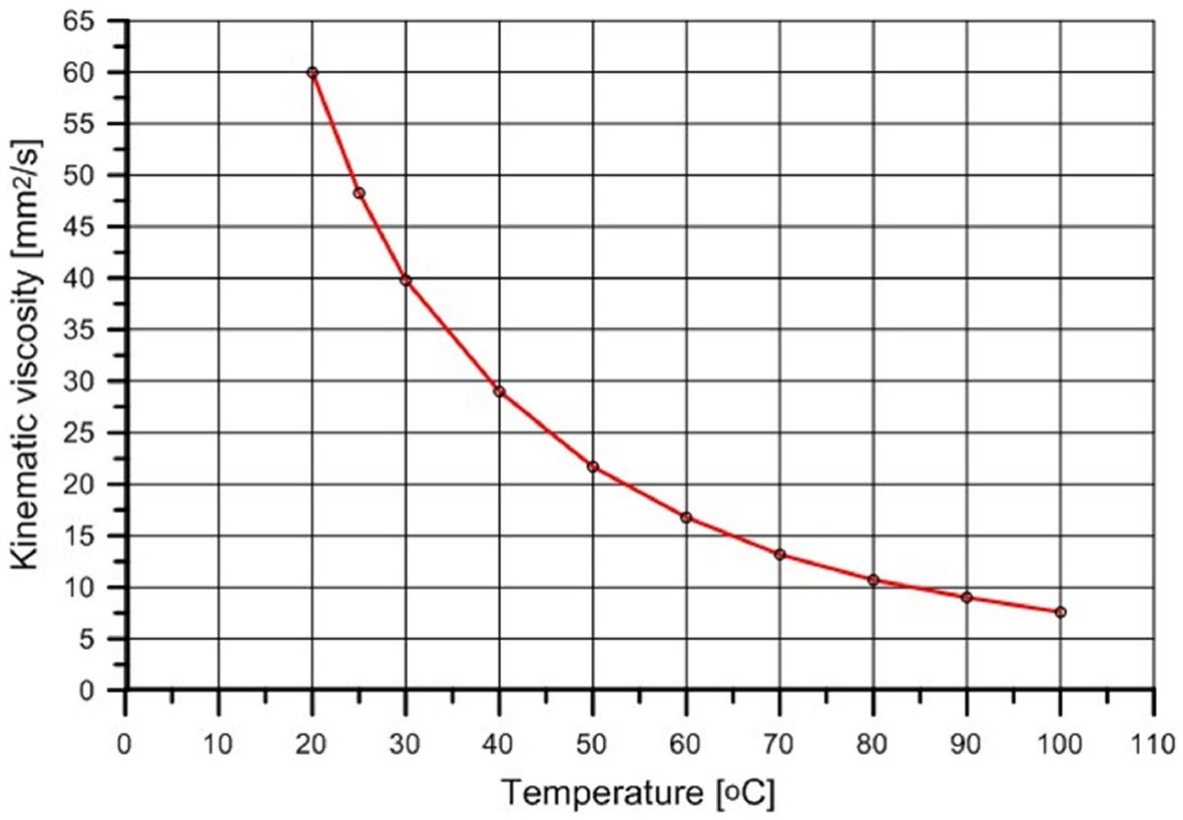
Raw Camelina sativa oil kinematic viscosity vs. temperature.

**Figure 2:**
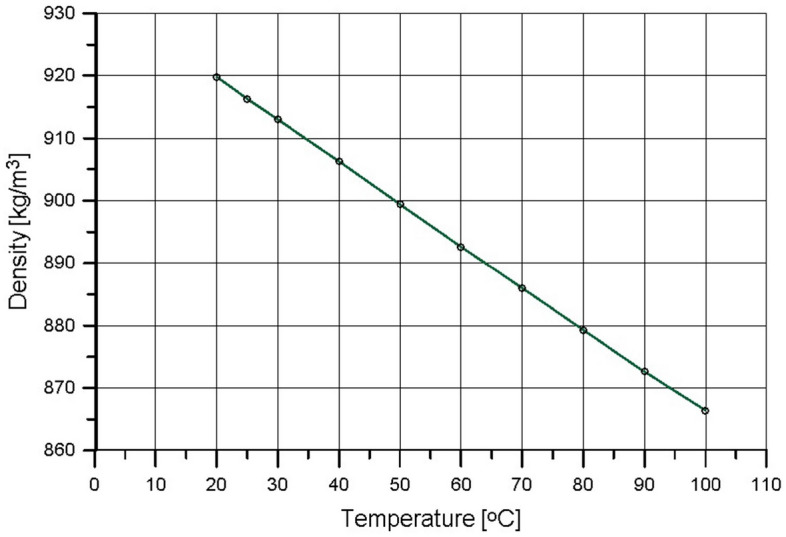
Raw Camelina sativa oil density vs. temperature.

The Camelina sativa oil used in the experiment was obtained from a winter variety of plants produced in Poland. The oil had been purchased from the herbal farm. It was obtained by cold pressing. During the process, the temperature of the oil reaches approximately 45 **°**C. Then, the oil was subjected to 3 days of sedimentation. The experiment described in the paper included the examination of the kinematic viscosity and density of the oil used in the engine tests at different temperatures. The viscometer type Stabinger SVM 3000 Anton Paar was used to determine these parameters. The results are presented in Figs. 1 and 2.

The fuel temperature measured in the common rail during the engine run was 70 **°**C. The experimentally determined kinematic viscosity of raw Camelina sativa oil at 70 **°**C was 13 mm^2^/s (still much higher than the kinematic viscosity of diesel oil which is 2.35 mm^2^/s at 40 **°**C). It means that Camelina sativa oil injected into the engine cylinder could create the air–fuel mixture more easily than vegetable oils injected by conventional, low-pressure systems at lower temperatures. It is also evident that the spray and its atomisation parameters were different for the same injector nozzle used in the experiment for both fuels^10^.

Figures 1 and 2 show how the fuel system can modify fuel properties, realising its preheating. The comparison of the properties of raw Camelina sativa oil and diesel oil is presented in Table 1.

**Table 1:** The comparison of raw Camelina sativa and diesel oil properties.

Properties	Unit	Camelina sativa	Diesel oil
Low heating value	MJ/kg	38.7	43.2
Cetane number	–	42.3	55.0
Liquid density at 15 °C	kg/m^3^	923	831
Kinematic viscosity at 40 °C	mm^2^/s	29.0	2.35
Sulfur content	mg/kg	13.8	10.0
Water content	mg/kg	720	43.8
Ash	kg/kg	0.003	0.01
Flash point	°C	> 220	55

*Source* Authors, data from Refs.^32,51^.

The tests were carried out on AVL experimental CI engine with a typical compression ratio of 17:1, equipped with a common rail system (Table 2) for engine speed n = 1500 rpm (yielding generator frequency f = 50 Hz). For each tested point, cylinder pressure diagrams from 100 cycles were registered, and the parameters that describe the combustion process were calculated. The exhaust gas analysis was carried out using AVL SESAM i60 F.T. analyser, which creates an infrared broadband spectrum that is used to detect all spectrum information of the exhaust gas sample simultaneously.

**Table 2:** Engine characteristics.

Model	AVL 5402
Type	Four-stroke, naturally aspirated, direct injection
Displacement (cm^3^)	511
Number of cylinders	1
Bore/stroke (mm)	85.01/90.00
Compression ratio	17
Fuel system	Common rail
Maximum power (kW)	ca. 16
Maximum speed (rpm)	4200
Inlet valve open/close (CA deg)	346/586.5
Exhaust valve open/close (CA deg)	128.5/376.5
Valve overlap (CA deg)	80
Nozzle type	DLLA 162 P 2160

*Source* data from Ref.^52^.

In the case of an experiment in which the same parameters are compared, repeatability of measurements is crucial. The calculation of percentage equipment variation (%EV), which is a measure of the repeatability of the measurement experiment, is described in Ref.^53^. Its value for the AVL measurement system used in the test is %EV = 0.4% (the measurement system is considered appropriate for the specific task if its %EV is below 10%). The maximum error of the pressure measurement in the combustion chamber is Δ_p_ =  ± 0.4%. It was calculated as a square root of the sum of squares of pressure sensor linearity error (δ_ps_ =  ± 0.3%), charge amplifier linearity error (δ_al_ =  ± 0.1%), amplifier noise (δ_an_ =  ± 0.2%) and analogue-to-digital converter linearity error (δ_adl_ =  ± 0.15%). The fuel consumption measurement accuracy (R_a_) for the Bronhorst Cori-Flow flowmeter used in the experiment is calculated using the formula R_a_ =  ± (0.2%Rd + 8 g/h). Maximum error of fuel consumption is ΔG_h_ =  ± 0.77%. In the experiment, the engine speed was constant (n = 1500 rpm ± 10 rpm). The maximum error for λ measurement (Lambda Meter LA4) is Δλ = ± 1.0%. The calibration process of AVL SESAM i60 F.T. analyser and the rest of the measurement equipment was carried out following the manufacturer's recommendations.

The combustion process in CI engines strongly depends on the mixture composition. The engine tests were performed for excess air coefficient λ = 1.1, 1.28, 1.52 and 2.12. After completing a series of tests to achieve a high BMEP of the engine, the fuel injection parameters were selected. For all examined points of the engine run, the Camelina sativa dose was divided into a constant preliminary dose (2.4 mg/cycle) and the main dose, depending on the excess air coefficient and engine load. The injection timing of the preliminary dose of Camelina sativa oil was constant (16 deg BTDC). The injection timing of the main dose was adjusted to get the maximum engine load for the selected excess air coefficient (λ) and to minimise the rate of cylinder pressure rise (dp/dα < 7 bar/deg) to eliminate the engine knock. The injection pressure of the fuel was set at p_in_ = 65 MPa.

The results obtained for raw Camelina sativa oil were compared to diesel oil for the same values of excess air coefficient (λ), the same injection strategy and the same injection pressure (p_in_). Because of the significant difference in the combustion process and, as a result, significantly lower engine thermal efficiency, the injection parameters for diesel oil were slightly modified to check the possibility of obtaining higher engine efficiency for the strategy utilised for Camelina sativa oil. The mass of diesel oil injected as a preliminary dose was reduced to 1.0 mg/cycle. Also, its injection timing was changed to 15 deg BTDC (16 deg BTDC for CS).

In order to illustrate the injection process, the voltage signal from the common rail injector was registered. It is shown in the volumetric heat release rate (VHRR) and cylinder pressure (p) diagrams presented in the paper.

## The results

**Figure 3:**
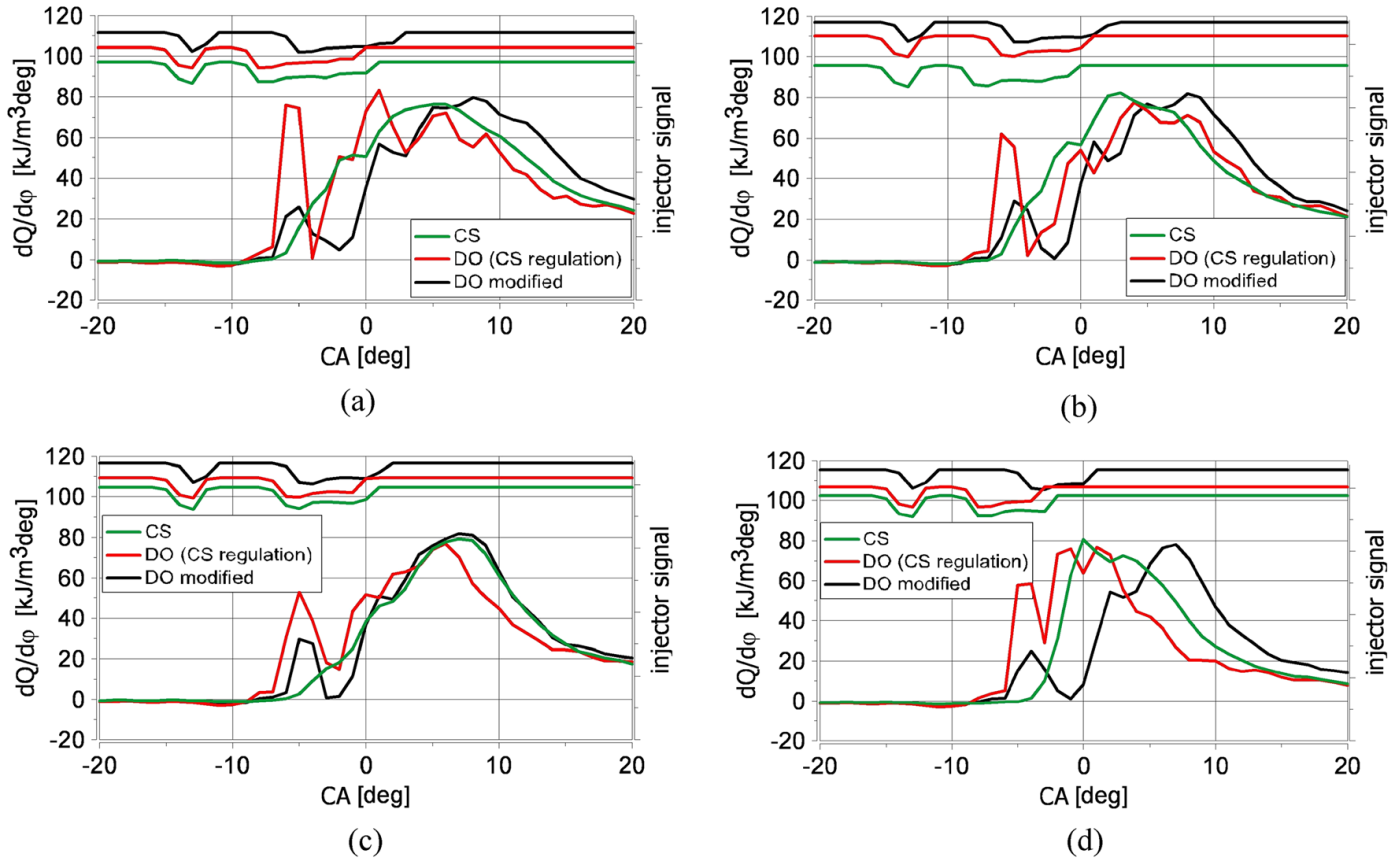
Volumetric heat release rate diagrams for the same injection strategy applied for raw Camelina sativa oil (CS) and diesel oil (DO (CS regulation)) and for modified injection strategy for diesel oil (DO modified): (**a**) λ = 1.1, (**b**) λ = 1.28, (**c**) λ = 1.52, (**d**) λ = 2.12. The injector signal diagrams are above the volumetric heat release rate diagrams.

**Figure 4:**
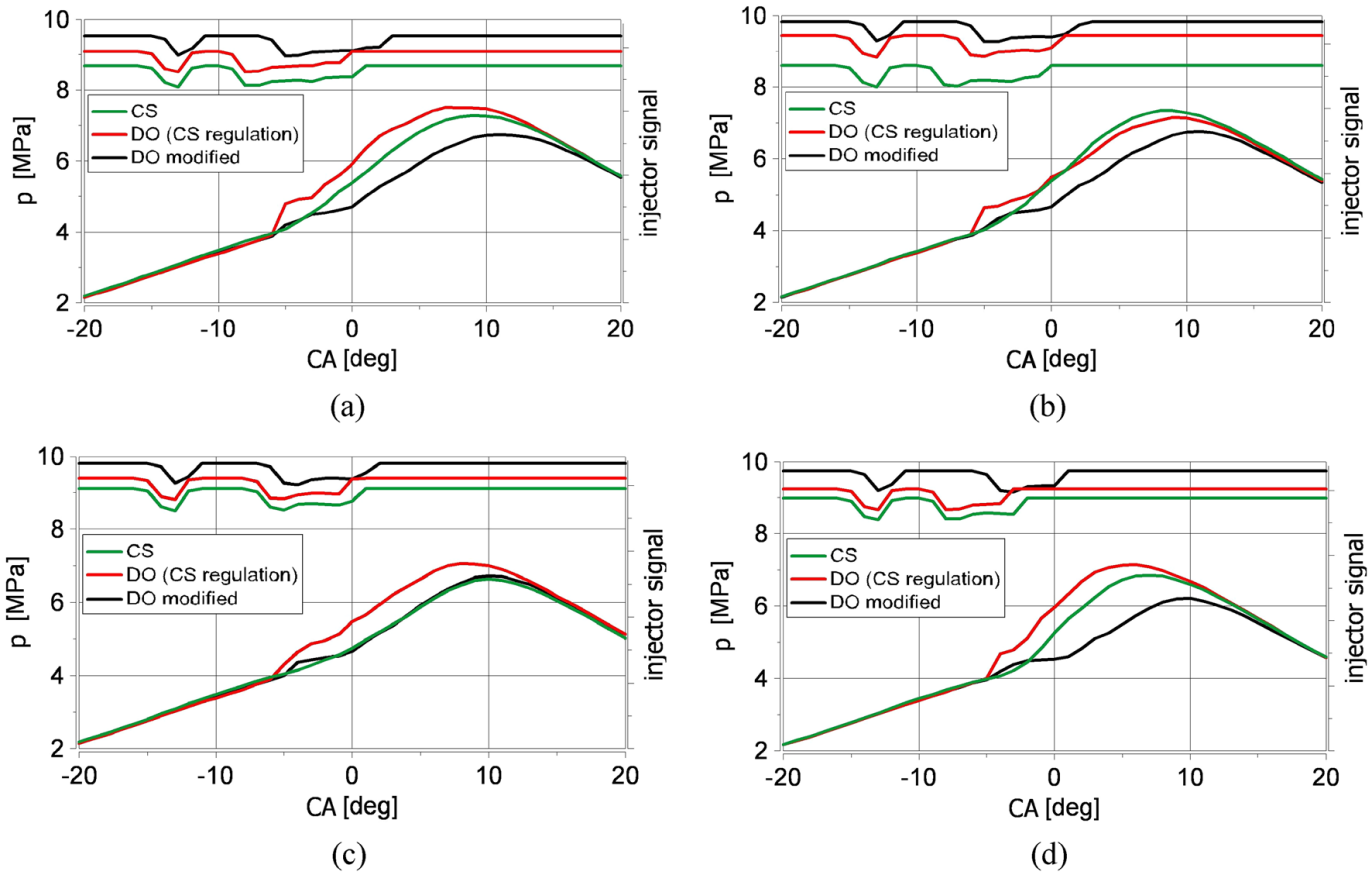
Cylinder pressure diagrams (fragments) for the same injection strategy applied for raw Camelina sativa oil (CS) and diesel oil (DO (CS regulation)) and for modified injection strategy for diesel oil (DO modified): (**a**) λ = 1.1, (**b**) λ = 1.28, (**c**) λ = 1.52, (**d**) λ = 2.12. The injector signal diagrams are above the volumetric heat release rate diagrams.

**Figure 5:**
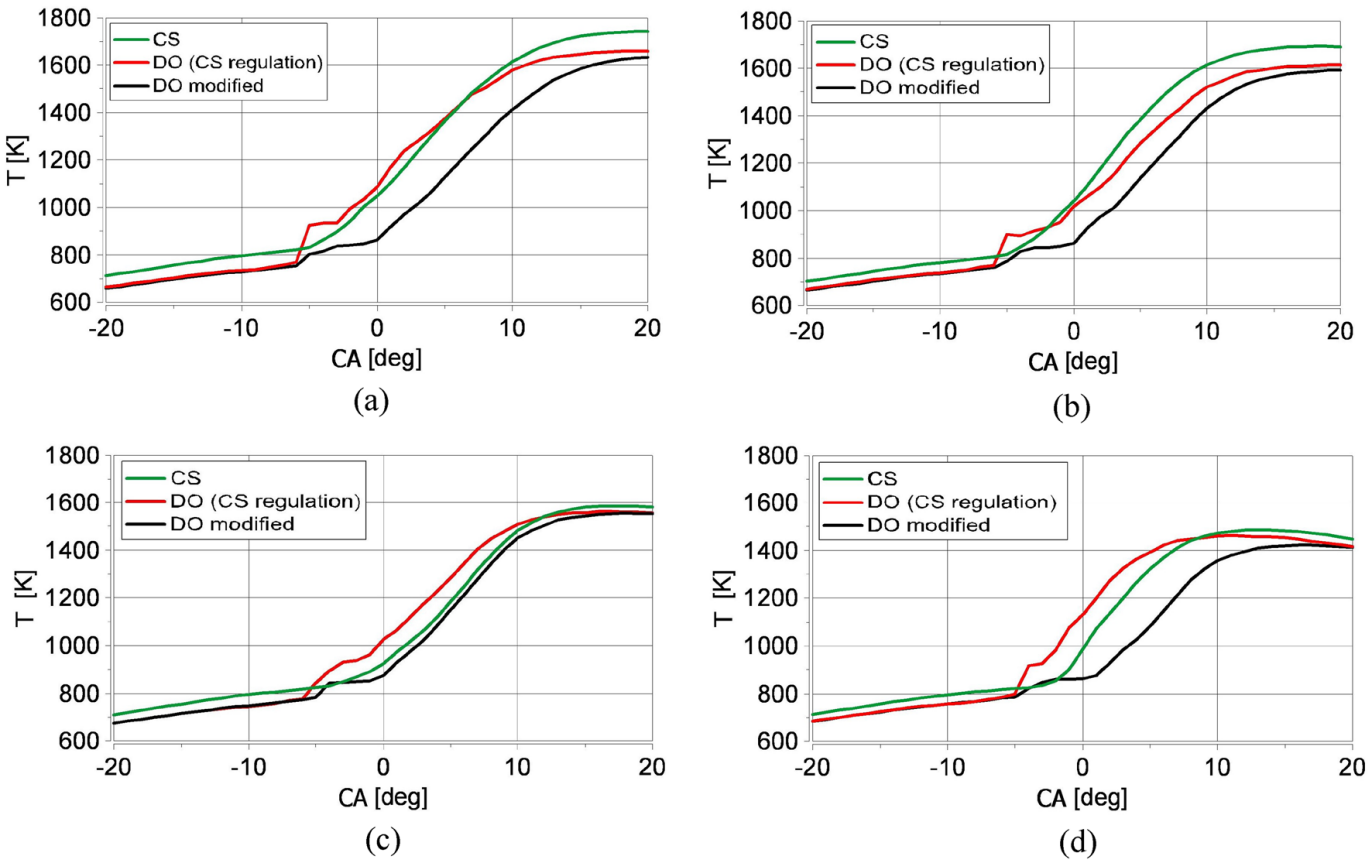
In-cylinder temperature diagrams for the same injection strategy applied for raw Camelina sativa oil (CS) and diesel oil (DO (CS regulation)) and for modified injection strategy for diesel oil (DO modified): (**a**) λ = 1.1, (**b**) λ = 1.28, (**c**) λ = 1.52, (**d**) λ = 2.12. The injector signal diagrams are above the volumetric heat release rate diagrams.

The volumetric heat release rate diagrams calculated for raw Camelina sativa oil for different excess air coefficients (λ) are presented in Fig. 3. Additionally, measured cylinder pressure (Fig. 4) and calculated in-cylinder ambient temperature are shown in Fig. 5. For comparison, the diagrams obtained for raw Camelina sativa oil are compiled with those received for diesel oil fueling (for the same injection strategy as for Camelina sativa oil and its slight modification).

**Figure 6:**
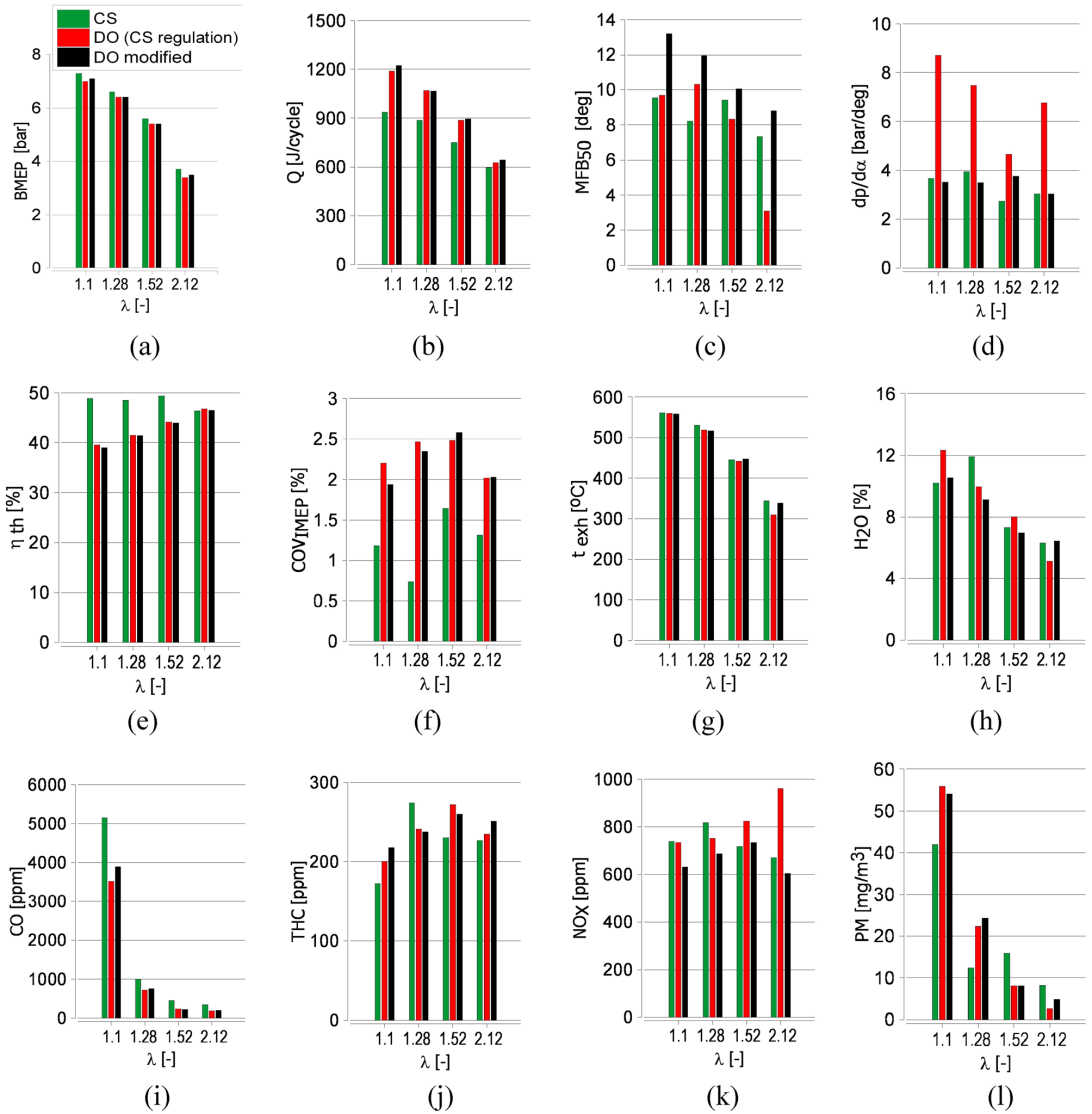
Measured and calculated engine parameters for the same injection strategy applied for raw Camelina sativa oil (CS) and diesel oil (DO (CS regulation)) and for modified injection strategy for diesel oil (DO modified) for different λ: (**a**) BMEP, (**b**) Q_c_, (**c**) MFB50, (**d**) dp/dα, (**e**) η_th_, (**f**) COV_IMEP_, (**g**) t_exh_, the volumetric share of: (**h**) H_2_O, (**i**) CO, (**j**) THC, (**k**) NO_x_ in exhaust gases, (**l**) PM concentration in exhaust gases.

The combustion mechanism is significantly different for the same injection strategy used for both examined fuels (VHHR green curve in Fig. 3), and the character of differences are the same for all tested mixture compositions. The denser and more viscous CS oil started to burn later, and a diffusive mixing rate controlled its combustion. The effect of the initial dose was visible with a faster-growing course of the VHHR diagram, which may suggest a small share of the combustion phase in which chemical kinematics affects the course of heat release. The volumetric heat release rate slowed down slightly after the injection of the main dose, resulting from the heat absorption needed to evaporate the injected fuel. This phenomenon was observed around TDC (Fig. 3). Further heat release proceeded smoothly according to the diffusion mechanism. The leaning of the mixture only shifted the start of combustion. The injection timing allowed 50% of the fuel to be burned between 7 and 9 deg after TDC (Fig. 6c). The combustion mechanism results in the engine’s high thermal efficiency (Fig. 6e). Still, it contributed to a noticeable NO_x_ emission rise in comparison to others tested points of engine run (Fig. 6k).

The combustion of diesel oil injected according to the strategy applied for Camelina sativa oil resulted in premixed charge compression ignition (PCCI). Compared to Camelina sativa, the ignition delay was shorter, advancing the rich premixed spike from the late main dose injection. It was apparent for lean mixtures (Fig. 3d). Diesel oil started burning just after the end of the preliminary dose injection at a lower temperature than for Camelina sativa and was primarily governed by chemical kinetics. Then the diffusive mechanism^54^ is visible (red curve in Fig. 3). For all examined λ the volumetric heat release rate curves for diesel oil, a characteristic hump in the heat release rate curves appeared (Fig. 3a–d). The premixed peak of VHRR diagram for diesel oil is nearly as high as the mixing-controlled one. In this case, the stratified and quasi-homogeneous phases of combustion are separated. This phenomenon caused a sudden increase in ambient temperature in the engine cylinder visible for all tested λ (Fig. 5a–d) and an accelerated increase in pressure in the combustion chamber Figs. 4a–d and 6d.

The combustion mechanism of diesel oil for the same injection strategy as for Camelina sativa did not result in high engine thermal efficiency as for Camelina sativa oil burning because a significant amount of heat was released before TDC (Fig. 6c). The slight change of injection parameters for diesel oil (reduction of preliminary dose and the main dose retardation) allowed to shift the ignition delay and to minimise the value of the rate of cylinder pressure rise below 4 bar/deg but the mechanism of combustion remained the same (DO modified in Figs. 1, 2, 3). It was visible that the diesel oil injection parameter demanded further adjustment to raise engine thermal efficiency. The search for the optimal diesel fuel strategy is not this article’s subject. Still, testing two similar injection strategies for diesel oil showed the differences in the heat release process resulting from the different properties of the fuels. It showed how the injection strategy, optimal from the point of view of performance and engine thermal efficiency for Camelina sativa oil, cannot be used to supply the engine fueled with diesel oil. In the experiment, the amount of diesel oil provided to the engine cylinder per cycle was selected to maintain the same excess air coefficient (λ) for both fuels. As a result, the energy supplied to the engine cylinder per one cycle (Q_c_) with Camelina sativa was lower than for diesel oil fueling for the same λ (Fig. 6b). But the higher thermal efficiency obtained for Camelina sativa fueling (Fig. 6e) allowed to receive nearly the same BMEP for both fuels.

The emissions measurement gave comparable results for both fuels (Fig. 6i–l). There was one exception—CO emission for Camelina sativa fueling was much higher for λ = 1.1. The exhaust gas temperature and water content were comparable and depended on mixture compositions (Fig. 6g,h). Also, for both fuels, cycle-by-cycle variations of indicated mean effective pressure (CV_IMEP_) remained acceptable for all λ values (Fig. 6f). Still, the factor remained lower for Camelina sativa for all examined points.

**Figure 7:**
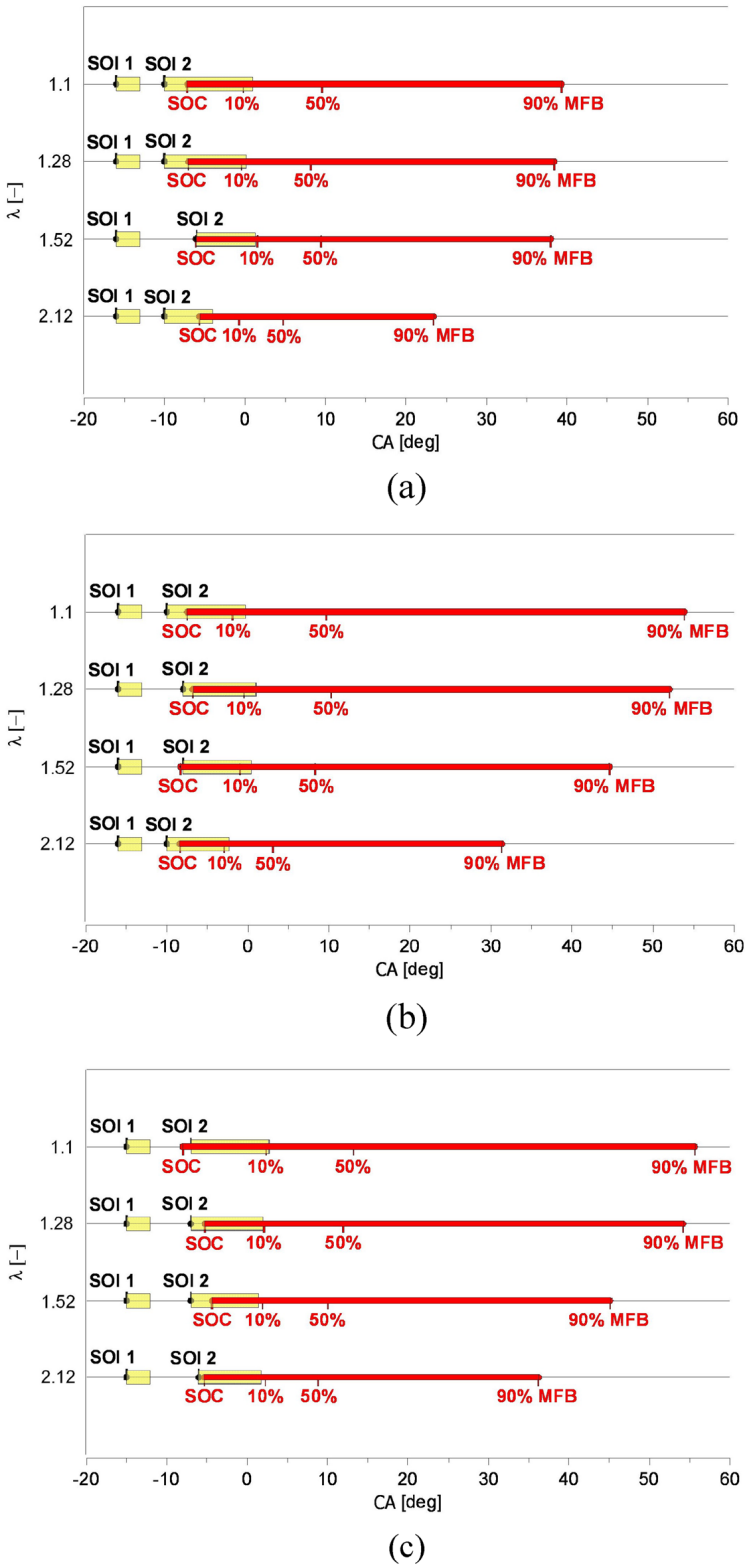
The comparison of some characteristic parameters of the injection and combustion process of raw Camelina sativa oil and diesel oil (two injection strategies) for different excess air coefficients (λ). (**a**) Raw Camelina sativa oil, (**b**) diesel oil (the same injection strategy as for raw Camelina sativa oil), (**c**) diesel oil (modified injection strategy).

Figure 7 compares some characteristic parameters of the injection and combustion process of both tested fuels for different λ (the yellow lines describe the injection process, but the red line illustrates the length of the combustion process). From the injection process’s point of view, the fuel’s temperature is crucial. Increased fuel temperature (70 °C) in the fuel system rail and high injection pressure (65 MPa) realised in the system confined self-ignition delay, which was only 2–3 CA deg longer than obtained for diesel oil (Fig. 7a–c). Despite later ignition, the combustion process of Camelina sativa oil took less time than diesel oil (Fig. 6c). Its 50% mass burnt period (50% MFB) is nearly the same for λ = 1.1, 1.28 and 1.52 (Fig. 7a). It contributes to high BMEP (Fig. 6a) and engine thermal efficiency η_th_ (Fig. 6e) for different mixture compositions. The tests confirmed the possibility of getting high engine performance for vegetable oil fueling^49^.

## Conclusions

Applying the CR system for raw Camelina sativa oil fueling allowed for obtaining high engine loads with high engine thermal efficiency. The experiment showed the benefits of the CR system application for injecting vegetable oil connected with the rising injected fuel temperature and possible modification of the heat release process by adjusting the injection strategy. The comparison of the volumetric heat release rate for the same injection strategy applied for Camelina sativa and diesel oil showed differences in the combustion process resulting from tested fuels’ different physical and chemical properties.

In the experiment, we didn’t deal with the durability and reliability of the engine fueled in the proposed way. Only some possible problems related to the practical application of vegetable oils as a fuel were mentioned in the text. The results described in the paper encourage further research work in this field.

The energetic transition demands time. Camelina sativa can be applied for well-known power generation technology, CI engine. Its utilisation as a fuel would benefit the economy and agriculture. Increasing cultivation and the associated reduction in the price of oil produced from Camelina sativa for local energy production can be a way to use large areas of wasteland. It is particularly important in agroecosystem creation in climate change time.

## Data Availability

The datasets used and/or analysed during the current study available from the corresponding author on reasonable request.

## List of symbols

BMEP: Break mean effective pressure
BTDC: Before top dead centre
CA: Crank angle (deg)
CFPP: Cold filter plugging point
CI: Compression ignition
CO: Volumetric share of carbon monoxide in exhaust gases (%)
COV_IMEP_: Coefficient of variation of the indicated mean effective pressure (%)
CR: Common rail
DO: Diesel oil
dp/dα: Cylinder pressure rise rate (bar/deg)
H_2_O: Volumetric share of water vapour in exhaust gases (%)
HCCI: Homogeneous charge compression ignition
IMEP: Indicated mean effective pressure (bar)
m: Mass (kg)
MFB50: Crankshaft position where 50% of fuel mass burned over an engine cycle is reached (deg)
NO_x_: Volumetric share of nitric oxides in exhaust gases (%)
p: Cylinder pressure (bar)
PM: Particulate matter concentration in exhaust gases (mg/m^3^)
PCCI: Premixed charge compression ignition
RCCI: Reactivity controlled compression ignition
Q_c_: Energy provided to the engine cylinder with the fuel per one cycle (J/cycle)
SOI: Start of injection (deg)
T: In-cylinder temperature (K)
TDC: Top dead centre
t_exh_: Exhaust gases temperature (°C)
THC: Volumetric share of total hydrocarbons in exhaust gases (%)
VHRR: Volumetric heat release rate (kJ/m^3^deg)
λ: Excess air coefficient (–)
η_th_: Engine thermal efficiency (–)
